# Practical Clinical Remarks—On Tetanus

**Published:** 1860-11

**Authors:** Frederick C. Skey

**Affiliations:** Surgeon to the Hospital


### PRACTICAL CLINICAL REMARKS,

### DELIVERED AT ST. BARTHOLOMEW'S HOSPITAL.

BY FREDERICK C. SKEY, ESQ., F. R. S., SURGEON TO THE HOSPITAL.

### ON TETANUS.

GENTLEMEN: One of the most mysterious amongst the numerous consequences of local injury is tetanus—a condition of the system that has hitherto for the most part baffled the highest efforts of human skill to arrest or cure. It cannot be denied that every case of tetanus has not proved fatal. On the contrary, cases of no ordinary severity have recovered through the instrumentality, or at least consequent on the employment of remedies, but which presumed remedies having failed to obtain relief in other and similar cases, warrant the not unreasonable conclusion that recovery in the case or cases in question was not attributable to the agent adopted. I remember a case of tetanus treated by Mr. Abernethy in St. Bartholomew's Hospital with calomel and jalap, in doses of from one grain to five. The case was a severe one, and arose, if I recollect rightly, from a wound about the hand. The man's bowels were greatly constipated. The purgative, given every three hours in repeated doses, at length operated freely, producing several most offensive evacuations. Relief from the spasm followed, and the man recovered. Mr. Abernethy, from that time, henceforth, in his surgical lectures, recommended to his pupils the adoption of the particular agent he had himself found so successful in the above case, attributing the disease to a constipated condition of the alimentary canal. And yet tetanus is a rare affection, while constipation in every form and degree is of common occurrence. How far can the presence of a wound or other form of local injury give a specific or at least a peculiar form to the constipation consequent on it? But it is notorious that purgatives do not cure tetanus; and that they have not the confidence of the profession, is obvious from the general resort to remedies of a directly opposite tendency. Indeed, it would be difficult to point at the present hour to any agent that does command professional confidence, whether sedatives, anti-spasmodics, counter-irritants, or any other therapeutic agent. Amputation of the affected limb, though occasionally resorted to with recovery, has much more frequently failed. Much was expected from the agency of chloroform. Many besides myself have proved its incompetency to any ultimate benefit. Temporary good of a very remarkable kind cannot have escaped the observation of any surgeon who has employed it freely in these distressing cases. It is something to allay spasm and its concomitant pain, to lower the pulse to nearly its natural standard, to restore the natural respiration, and to recall the ordinary expression to the distorted features. All this is done by chloroform, which however, while it draws a temporary mask over the symptoms, allows the under-current to flow with an insidious but certain force till it reaches its crisis in death. I have elsewhere given the details of a case of tetanus, in which under the administration of chloroform, a pulse of 130, coupled with rapid breathing, fell to the standard of health, as did the respiration, for a period of some eighteen hours, with the single interval of about half an hour, during which the administration of the agent was suspended, when the former symptoms instantly returned. Nor was the influence of the chloroform on the spasmodic condition of the affected muscles less remarkable. How conclusively do these facts point to the real nature and seat of the disease as centered in the nervous system, and prove that the circulating system is but secondarily involved! No sooner is the nervous centre sent to sleep than the disease appears as suddenly to cease. Notwithstanding this improvement, so deceptive and insidious, it is obvious that morbid actions predominate, and I doubt whether such temporary improvement, plausible and indeed marvellous as it is, can be said to indicate an arrest of the disease in however slight a degree.

A boy, aged fourteen, was brought into St. Bartholomew's Hospital, with a hand greatly mutilated by machinery. The house-surgeon decided, on consideration—and I thought wisely decided—to make the attempt to restore the hand. For twelve days he progressed favorably. On the thirteenth, he complained of stiffness in the back of his neck. When I saw him, at the expiration of some hours, spasm of the muscles had extended to the chest and abdomen; but none of the muscles were severely or painfully contracted. Still the signs were distinct. The boy was the subject of tetanus; and as I had never had a successful case, I was the more anxious to adopt some more promising agent than any I had hitherto employed. With this view I determined to test the efficacy of the woorara poison, which had been employed with some effect by Mr. S. Wells, in three cases of tetanus. By the kindness of Mr. Savory, who had a small quantity of the fresh poison, I was enabled to obtain sufficient for the purpose. We dissolved two grains in one ounce of distilled water. The boy being placed under the influence of chloroform, I amputated the hand immediately above the wrist-joint. My intention was to have applied two drops of the solution to the wound, and, observing the consequences, to have increased the quantity applied according to the effect produced. On recovering his consciousness, it was obvious that what had been done for the boy, whether the removal of the hand or the administration of chloroform, had been in the direction of good. The pulse had fallen from 135 to 100, and the rigidity of the muscles was greatly diminished and continued so. The pain was also reduced and the countenance was more tranquil. He could open his mouth to an extent sufficient for the introduction of food. The rigididity of the muscles was by no means great, nor did he suffer the degree of pain usually attendant on severe cases of tetanus. It is very true that the sterno-mastoids, pectorals, abdominal muscles, and, indeed, the muscles of his trunk generally, were involved; but their contraction was comparatively slight, and certainly was unproductive of considerable pain. The use of the woorara was postponed, and I ordered the boy tincture of opium, ten minims; compound spirit of ammonia, half a drachm; brandy, half an ounce; every two hours. On the following day, I found he had had a tolerably good night; his pulse had slighly risen in frequency; the rigidity of the muscles had not increased; on the contrary, it appeared somewhat lessened. His countenance was more anxious, which appeared rather attributable to mental than to bodily suffering, for he had only recently become cognizant of the removal of his hand. By two o'clock on that day, the boy had consumed four ounces of brandy, half an ounce of spirit of ammonia, and eighty drops of laudanum. Still I had no excuse for the trial of the woorara. Doubtless the boy was in a dangerous condition; but it was obvious that the condition was not attributable to the morbid contraction of the muscular system, but to something beyond it. His bowels were constipated. I ordered him calomel and jalap, castor oil enemata of turpentine, croton oil in no insignificant doses, but I failed to produce the desired result up to the time of his death. In other respects, the same treatment was continued. On the third and fourth days his condition was not materially changed. He took food without difficulty, as also his medicine at the required intervals. The ammonia was increased to drachm doses. On the fourth day his pulse had increased to 135, and his countenance was more pallid. The rigidity of his muscles had not increased in any positive degree, and those of the extremities were entirely free from spasm. On the morning of the fifth day, he had taken, without marked effect on his system for good or for evil, about forty-eight ounces of brandy, one ounce of tincture of opium, and four ounces of spirit of ammonia. And we may form some judgment of the severity as well as the prostrating influence of the disease under which this poor boy was laboring, when we consider that such an enormous power was inadequate to produce the smallest impression on his system. On the last visit I paid, except that he appeared some weaker,

I cannot say that he was positively worse. He could open his mouth sufficiently wide for the introduction of nourishment, and he took food freely through the greater part of his illness. His pulse was a shade weaker, but not materially so. His spasms were not severe nor considerable, and assuredly they were not a source of pain; of this the boy gave me full assurance on many occasions. On the evening of the fifth day he died. His body was not examined.

If the nature of the disease we call tetanus could be ascertained by dissection, we should have acquired this knowledge long ago; but, so far as I know, we have obtained no information by such means, except negatively, on which to found a theory, or to obtain a guide to practice. The total amount knowledge consists in this: that at varying intervals after the occurrence of more or less violent local injury, and occasionally—but in our latitude very rarely—independently of injury, we observe rigidity of the muscular system, commencing with the muscles of the jaw and extending to the neck, trunk, and finally to the extremities; that it is accompanied by great pain in the contracted muscles, a quick pulse and respiration, and constipated bowels; and that it terminates in from two to six or eight days in death. Such is nearly the sum total of our knowledge of tetanus, and it may be doubted whether we are even now occupied in the right path of inquiry. We fix our attention on the local spasm of the muscular frame, as though that condition of the system constituted the essence of the disease, of which it is only a symptom. We rather search for remedies that will abate spasms, than for such as will attack its cause. The above case exemplifies negatively this fact. The boy died of tetanus, but not of spasm. At no period of his case was the contraction of his muscles so rigid as to cause pain of a severe character. He complained of his arm; he suffered from a sense of illness; he desired to be let alone, and not to be disturbed; his expression betokened serious illness; but he did not die from pain nor spasm; he gradually sank from utter prostration of nervous power, which every remedy employed was incompetent to contend against. And it is by no means uncommon to observe, towards the close of life, that the most prominent symptom of the disease—namely muscular rigidity—gradually subsides, while the real disease advances towards its fatal crisis. In the whole range of disease, I do not recollect any evidence of the existence of what we term the nervous system more remarkable or more conclusive than is derived from the influence of anæsthetics in tetanus. How absolutely is the morbid influence of the nervous system upon the heart and respiratory system suspended by chloroform! and how suddenly, too! The pulse falls to its natural standard, the equilibrium of respiration is restored, all expression of suffering subsides under the reality of tranquil sleep. So complete is the external manfestation of health, that it would astonish a bystander, ignorant of the nature of the case, to be assured that in a few days, possibly in a few hours, it would terminate in death. Suspend the remedy, remove the influence of the anæsthetic agent, and the entire train of fatal symptoms starts, as it were, into existence—the pulse rises, the respiration is quickened in proportion, and the muscular spasm, with its usually inseparable companion, pain, become again dominant. Nor do we observe that the disease, to whatever extent it may have reached, is retarded or even diminished by the treatment. On the contrary, its progress is unremitting; and both the violence of the spasm and the severity of the pain are regenerated with an increase of intensity proportioned to the duration of time during which they have been suspended by anæsthetic agency. In the above case, I was fully prepared to test the curative properties of the woorara poison, as recorded by Mr. Wells and recent French writers; but I really had no excuse for its employment. The boy did not die either from or with spasm; and I cannot persuade myself that we shall ever discover in the woorara poison a curative property capable of rousing the vital powers, already oppressed by mysterious morbid actions going on within the frame. Still, I am quite prepared to acknowledge that all reasoning, more especially on a subject so obscure as tetanus, sinks in comparison with practical observation. Other authorties may view the subject in a different light; and it will prove no small accession to our knowledge should we discover in this or in any other untried agent, the power to arrest so hitherto fatal a disease as that which forms the subject of the above brief remarks.

